# Electron beam irradiation for the formation of thick Ag film on Ag_3_PO_4_†

**DOI:** 10.1039/d0ra03179h

**Published:** 2020-06-08

**Authors:** João Paulo de Campos da Costa, Marcelo Assis, Vinícius Teodoro, Andre Rodrigues, Camila Cristina de Foggi, Miguel Angel San-Miguel, João Paulo Pereira do Carmo, Juan Andrés, Elson Longo

**Affiliations:** Department of Electrical Engineering (SEL), University of São Paulo (USP) 13566-590 São Carlos Brazil; Department of Chemistry, INCTMN, CDMF, Federal University of São Carlos (UFSCar) 13565-905 São Carlos Brazil elson.liec@gmail.com; Department of Physical Chemistry, Institute of Chemistry, State University of Campinas-(UNICAMP) 13083-970 Campinas São Paulo Brazil; R&D Centre MicroElectroMechanics (CMEMS), University of Minho Campus Azurem 4800-052 Guimaraes Portugal; Department of Analytical and Physical Chemistry, University Jaume I (UJI) Castelló 12071 Spain

## Abstract

This study demonstrates that the electron beam irradiation of materials, typically used in characterization measurements, could be employed for advanced fabrication, modification, and functionalization of composites. We developed irradiation equipment using an electron beam irradiation source to be applied in materials modification. Using this equipment, the formation of a thick Ag film on the Ag_3_PO_4_ semiconductor is carried out by electron beam irradiation for the first time. This is confirmed by various experimental techniques (X-ray diffraction, field-emission scanning electron microscopy, Raman spectroscopy, and X-ray photoelectron spectroscopy) and *ab initio* molecular dynamics simulations. Our calculations demonstrate that, at the earlier stages, metallic Ag growth is initiated preferentially at the (110) surface, with the reduction of surface Ag cations forming metallic Ag clusters. As the (100) and (111) surfaces have smaller numbers of exposed Ag cations, the reductions on these surfaces are slower and are accompanied by the formation of O_2_ molecules.

## Abstract 2

This study demonstrates that the electron beam irradiation of materials, typically used in characterization measurements, could be employed for advanced fabrication, modification, and functionalization of composites.

## Graphical abstract

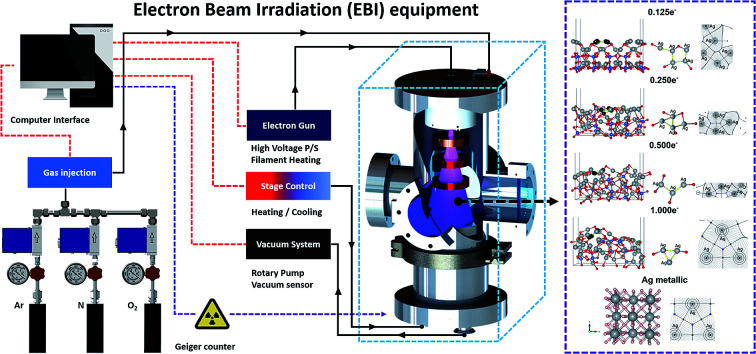

## 1. Introduction

Over the past years, microscopic techniques provided valuable insights into the compositions and structures of materials at the nano- and angstrom-levels.^1^ Among them, *in situ* transmission electron microscopy (TEM) is a powerful method for the investigation of atomic structures, dynamics, and diffraction patterns,^2^ and atomic-level analyses^3^ of various processes including phase/shape transformations,^1,4–7^ molten nanofluidic migration,^8^ and electromigration.^9^ The electron beam irradiation (EBI) in TEM could be also used to fabricate nanomaterials that cannot be obtained using conventional chemical and physical methods. It provides a versatile route to the syntheses of various types of nanoparticles and composite materials, of importance for the development of nanostructures. This synthesis method is advantageous as it satisfies the ‘‘green chemistry’’ objectives owing to the absence of chemical precursors (such as metal–organic substances), avoiding the use of toxic chemical reducing agents and surfactants or by products, which possibly adsorb onto the nanoparticle surface.^10–12^

The understanding of electron–solid interactions, which are the basis of these phenomena, is fundamental for the applications of the microscopy techniques in the syntheses, analyses, and modifications of materials. In such applications, energy is transferred from the energetic electrons to both electrons and atomic nuclei in the target materials, which leads to the generation of defects and changes in properties. The fabrication of various materials using EBI has attracted increasing attention.^10^ In addition, the finely focused electron beam could modify the compositions and structures of certain materials in a highly localized manner, which may be applied in nanotechnology. For example, EBI has been used to induce the formation of self-organized metallic nanostructures,^13^ crystalline Si nanodots in a SiO_2_ film,^14^ and hollow and toroidal NiO clusters^15^ and manipulate low-dimensional materials down to the level of single atoms.^16^

The electron beam processing technology has a high precision, high efficiency, advanced control characteristics, and high development speed. The application of EBI for the processing of nanostructures could provide various advantages, such as the adjustment of particle beam parameters, including the dose, energy, exposure time, and incident angle, at the specified location with a specific concentration. Several studies have elucidated the underlying mechanisms driving the electron-beam-induced syntheses.^17–20^ In particular, Kalinin *et al.*^21^ have summarized the progress in this field, from electron-beam-induced material transformations to atomically precise doping and multi-atom assembly, including the associated engineering, theoretical, and big-data challenges.

However, only a few recent studies have focused on the electron-beam- and probe-based fabrications and manipulations of two-dimensional materials in the electron microscope.^22–27^ The modelling of the electron beam dynamics has also attracted large interest.^28,29^ In this context, Bichoutskaia *et al.*^30^ summarized the experimental observations and analyzed the irradiation-induced processes in high-resolution TEM, while Knez *et al.* proposed a computational scheme of the electron-beam-induced dynamics in metallic nanoclusters.^31^

Our research group demonstrated that the EBI in TEM and/or field-emission scanning electron microscopy (FE-SEM) is a direct platform for the formation of Ag nanoparticles on the surfaces of different silver-based materials including Ag_2_WO_4_,^32–35^ β-Ag_2_WO_4_,^36,37^ β-Ag_2_MoO_4_ (ref. 37), and Ag_3_PO_4_ (ref. 38), β-AgVO_3_ (ref. 39) and Ag_2_CrO_4_ (ref. 40) crystals, and In and Bi nanoparticles from InP^41,42^ and NaBiO_3_, respectively.^43–45^ The formation of these metal nanoparticles–semiconductor composites provided enhanced surface functionalities and paved the way for the development of materials with bactericidal/antibacterial, antifungal, and antitumor characteristics for use in clinical sterilization, and catalysts, which is crucial for the sustainable development of energy-related catalysis, environmental sustainability, fine chemical industry, and biomedical applications.^46,47^ The *in situ* atomic-scale observation combined with realistic simulations based on first-principle calculations of nanomaterials enables the atomic-scale recording of events,^48^ which paves the way for novel syntheses and analyses of promising composite materials in material science and nanotechnology. This approach is advantageous as these phenomena occur under a high vacuum at room temperature.

The growth dynamics could be successfully investigated owing to the high spatial and temporal resolutions. Notably, the EBI can be regarded as a reductant for metallic nanocrystals, different from the reducing agents in the conventional solution chemical synthesis.

In particular, the sintering process of Ag nanoparticles by *in situ* electron beam irradiation in a transmission electron microscope supported by theoretical calculations.^49^ Furthermore, other research groups reported the electron beam sintering process for Ag and Pt nanoparticles^50^ and also for Au nanoparticles.^51^ However, it is still challenging to obtain scalable and viable productions of new materials with cost-effective and simple manufacturing procedures. According to the statement of Kroemer (Nobel Prize in Physics, 2000),“the interface is the device”,^52^ the studies on these material-based devices have not only contributed to the deeper understanding of their physical mechanisms but also provided a valuable platform for various applications, from electronics, optoelectronics, to energy and sensing.

**Fig. 1:**
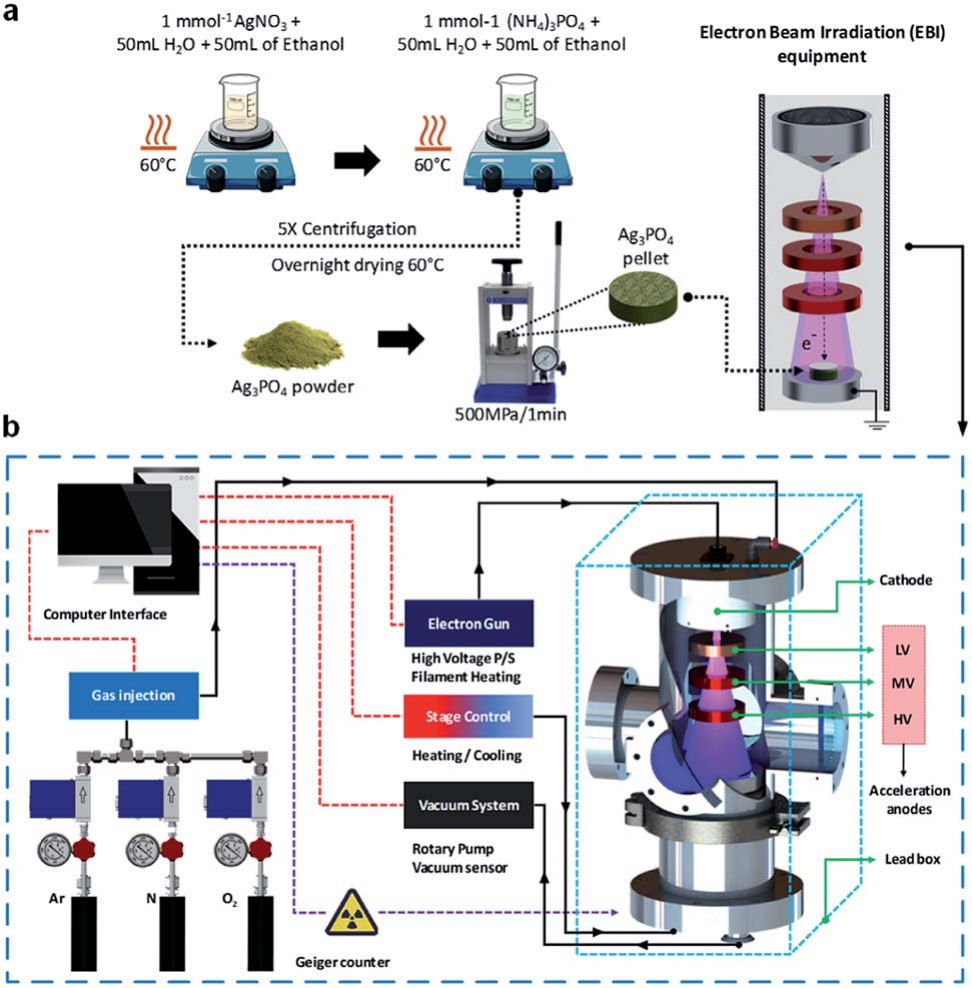
Schematic of the (a) synthesis and EBI of the sample; (b) developed EBI equipment.

Therefore, it is crucial to develop techniques enabling the design of materials with promising properties. In this study, we propose an EBI equipment for the irradiation of materials by an electron beam source, which satisfies the above requirements. In addition, for the first time, we investigate the formation of a thick Ag film on the Ag_3_PO_4_ semiconductor through detailed theoretical and experimental analyses. The structure of the thick Ag film was evaluated by X-ray diffraction (XRD) and micro-Raman (MR) spectroscopy. FE-SEM and X-ray photoelectron spectroscopy (XPS) were also employed for the characterizations of the structures. To complement the experiments results and aid the data interpretation, *ab initio* molecular dynamics (AIMD) simulations were performed to investigate the geometries, electronic structures, and properties of the pure and electron-irradiated Ag_3_PO_4_ structures. The geometries and electron density distributions were calculated to understand the effects of EBI and its relationships with the structural and electronic order–disorder characteristics of the Ag_3_PO_4_ lattice and surfaces. These results are valuable for understanding the effects of the different surfaces on their structures, energies, and electronic properties upon the EBI, which could extend the fundamental understanding of the effect of EBI on Ag_3_PO_4._Fig. 1 illustrate the synthesis process and the schematic of the developed EBI equipment.

## 2. Results and discussion

**Fig. 2:**
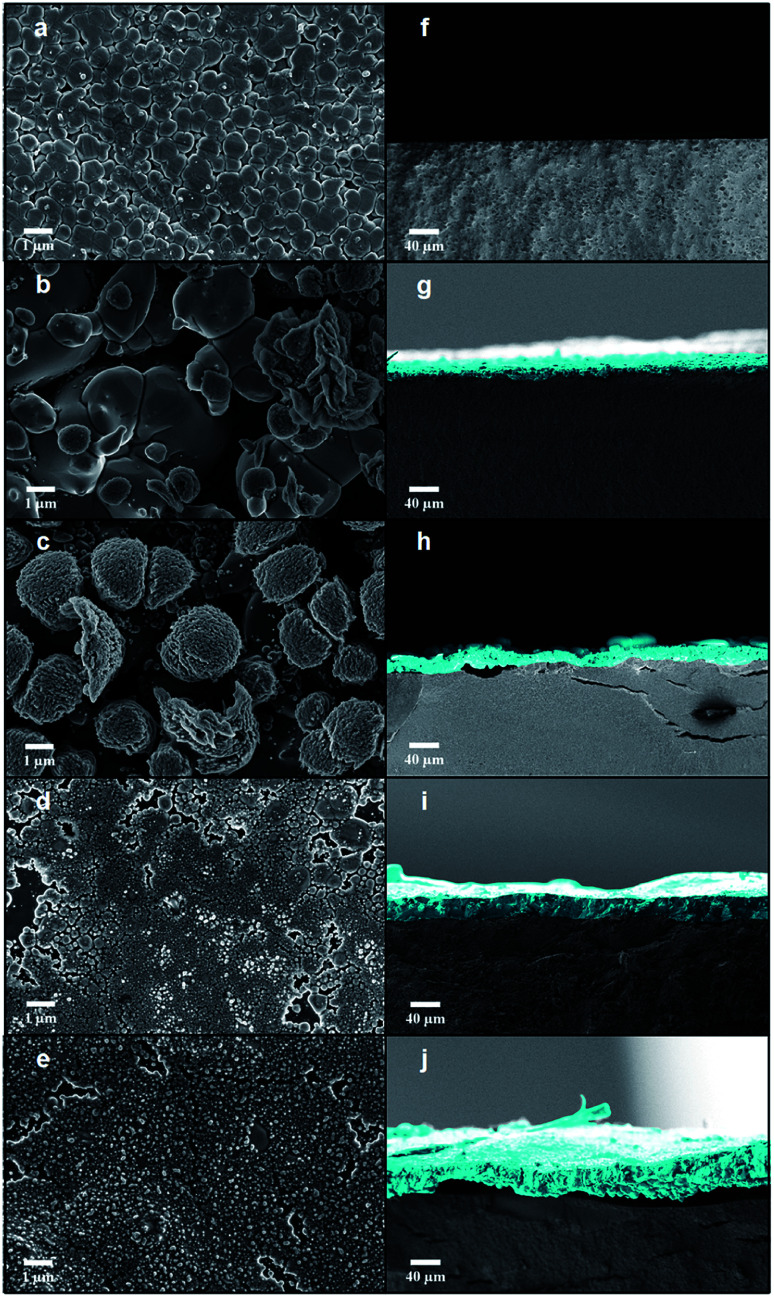
FE-SEM images of (a–e) the pellet surface and (f–j) the cross sections of all samples with different time irradiation 1, 2, 4 and 8 min, respectively.

FE-SEM images acquired after the EBIs of Ag_3_PO_4_ pellets at different times are shown in Fig. 2. Fig. 2a–e show FE-SEM images of the Ag_3_PO_4_ pellets surface. The Ag_3_PO_4_ pellet obtained without irradiation (Fig. 2a) exhibited an average particle size of 0.798 μm and high compaction. After EBI of the pellets for 1 min (Fig. 2b), an increased particle size was observed.

When high-energy electrons reach a material, transfer of energy occurs, which can transfer the electrons in the valence band to excited states.^53^ This enables various electronic and structural alterations in the material, such as the formation of vacancies, atomic displacements, formation of electron–hole pairs and excitons, decomposition, and crystallization.^34,54–57^ These changes are dependent on the interaction of the matter with the electron beam. Upon these changes, the diffusion processes can be favored owing to their high sensitivities to electronic and structural changes, provoking sintering processes. Therefore, according to Chernyavskii,^53^ the mass transport at high temperatures is accelerated in ionic structures owing to the heating of the electron beam with electron beam sintering. The sintering rate upon the EBI is dependent on the voltage, acceleration current, and vacuum of the equipment. Upon the increase in irradiation time, the formation of new structures (Fig. 2c) and subsequent formation of a sintered layer on the surface of the Ag_3_PO_4_ pellet are observed (Fig. 2i and j).

Cross-sectional images of the Ag_3_PO_4_ pellets are shown in Fig. 2f–j. When the Ag_3_PO_4_ pellet was irradiated for 1 min, a thick (6.857 μm) layer was formed on its surface, whose thickness increased with the EBI time (19.473, 27.329, and 38.504 μm for the times of 2, 4, and 8 min, respectively). Therefore, the penetration and the degree of modification of the pellet surface are dependent of the equipment parameters (e-beam energy, kV) and the layer thickness by the irradiation time.

In order to estimate the penetration depth of the incident electron beam (18 kV) through the formed Ag layers, calculated values can be obtained by the Kanaya–Okayama equation, which is usually employed in electron microscopy. According to this equation, considering an electron beam energy of 18 kV, the atomic weight and atomic number of silver atoms and the bulk density of silver instead the true density, the estimated value of penetration depth of the electron beam in our case is 38.7 μm. Despite the sintering process of Ag layer by electron beam irradiation, it can be observed in FE-SEM images and well expected that remains empty spaces between nanoparticles, *i.e.* pores. Since the electron beam passes through a layer formed by nanoparticles and hence pores, the bulk density has to be considered to the calculation, since it takes into account the empty space between nanoparticles, in contrast to the true density. In this sense, the maximum penetration depth of our incident electron beam in the Ag layer is about to 38.7 μm.

A common phenomenon that occurs in high-energy electron beam–matter interaction is the production of Bremsstrahlung X-rays as a consequence of decelerating electrons by interaction with electric field of nucleus and electronic shell of atoms. However, considering a partially kinetic energy decelerating or even the total kinetic energy (Duane–Hunt limit), the X-ray photons produced would provoke the photoelectric effect in the P and O atoms, and the photoelectrons in the *continuum* region could reduce the Ag^+^ ions. Nonetheless, due to thick Ag layer, the deceleration of electron beam would be near of Duane–Hunt limit, producing Bremsstrahlung X-rays of about 18 keV and hence photoelectrons with energies slight lower than 18 kV. According to this, the mean free path of these photoelectrons it would be approximately 1 nm, which is a relative short distance for an electron mobility to produce an Ag thick layer. Therefore, a possible mechanism for the layer formation is the injection of additional electrons in the Ag electronic structure by the electron beam irradiation.

The reduction of Ag^+^ ions for the formation of Ag^0^ nanoparticles or layers have been studied by several methods, such as photon irradiation,^58,59^ electron irradiation,^60^ and the use of reducing agents.^61,62^ Concerning to formation of metallic Ag nanoparticles or layers by visible light or ultraviolet irradiation, as widely reported by several works, the photoreduction process of Ag^+^ is usually conducted in aqueous medium and commonly requiring the use of additives.^63,64^ In these processes, the photoreduction mechanism arises from redox reactions that occurs in the solid–liquid interface, in which the liquid medium provides electrons to Ag_3_PO_4_ structure by oxidative reactions.

**Fig. 3:**
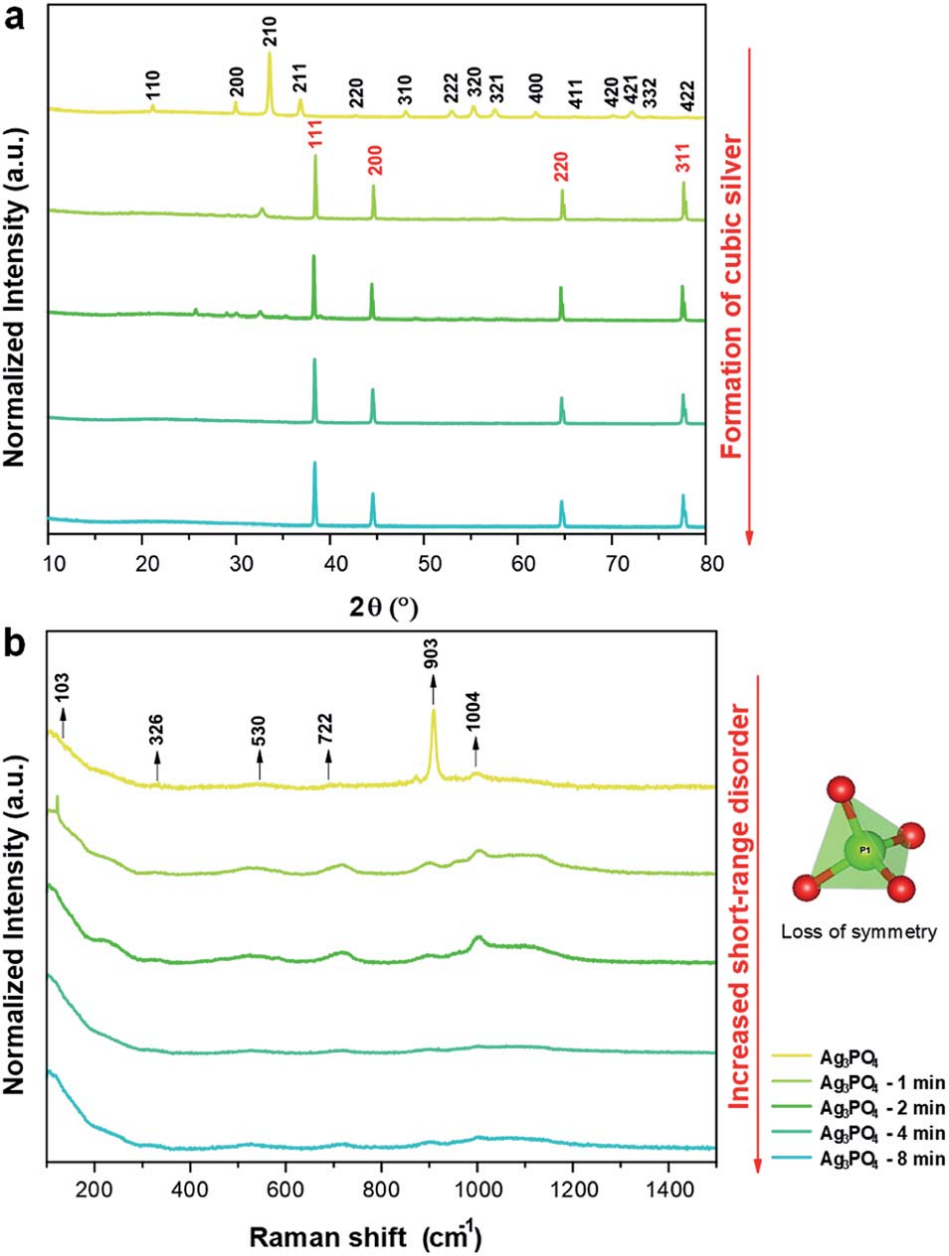
(a) XRD patterns of the samples irradiated for different times; (b) Raman spectra of the samples irradiated for different times.

XRD patterns are shown in Fig. 3a, which confirm the crystalline structures of the samples. The XRD patterns indicate that the unirradiated Ag_3_PO_4_ crystals have a cubic structure with a space group of *P*4̄3*n* with two molecules per unit cell (*Z* = 2), without secondary phases (Inorganic Crystal Structure Database (ICSD) no. 14000).^65^ This structure has two types of local coordination for both Ag and P atoms, corresponding to distorted tetrahedral clusters of [AgO_4_] and [PO_4_], respectively. When the pellets were irradiated for 1 min, the formation of an additional phase of metallic Ag was observed, corresponding to the cubic Ag (*a* = 4.085 Å) with the space group of *Fm*3̄*m* and four molecules per unit cell (*Z* = 4) (ICSD no. 44387 (ref. 66)). With the increase in irradiation time, a decrease in Ag_3_PO_4_ phase content and increase in metallic Ag content were observed, yielding only metallic Ag after 4 min.

The FE-SEM images are consistent with these results and explain the formation (at the atomic level) of the Ag layers on the surfaces of the Ag_3_PO_4_ pellets provoked by the EBI, as induced structural and electronic disorders associated with the reduction of the Ag cations of the [AgO_4_] clusters of the AgPO_3_ with the concomitant formation of metallic Ag.

Raman scattering spectroscopy of all samples were carried out to complement the XRD results and evaluate the short-range structural order/disorder. According to the group theory, the allowed representation for each of the corresponding Wyckoff positions in the structure of Ag_3_PO_4_ with the symmetry group of *P*4̄3*n* reveals 18 active modes in the Raman spectrum after the decomposition at point Γ (2A_1_ + 4E + 12T_2_). The obtained spectra of all samples are shown in Fig. 3b.

The samples exhibited modes at 103, 326, 530, 722, 903, and 1004 cm^−1^. The other modes were not observed owing to their low intensities or overlap with other bands.

The mode at 103 cm^−1^ corresponds to the E mode of translation/rotation of the [PO_4_] cluster.^38^ The mode at 326 cm^−1^ is related to the bending of the P–O bond.^67^ The mode at 530 cm^−1^ is related to the asymmetric bending of the T_2_ transition in the [PO_4_] cluster.^38,68^ The modes at 722 (T_2_), 903 (A_1_), and 1004 (T_2_) cm^−1^ are related to the stretching of the O–P–O bonds; the first two correspond to symmetrical movements, while the latter to asymmetric movements.^38,69^ With the increase in electron irradiation time, the number of Raman modes was reduced owing to the loss of symmetry of the [PO_4_] clusters of Ag_3_PO_4_. This is attributed to the destabilization of Ag_3_PO_4_ toward the Ag metal formation. Therefore, in addition to the XRD results and FE-SEM images, these results show that the irradiation with electrons considerably changed the initial order of the system to long-, medium-, and short-range. This disorder is attributed to the interaction of the electrons with the Ag_3_PO_4_, which increased the Ag–O bond distances in the [AgO_4_] cluster until the excess electrons could form the metallic Ag.^38^

**Fig. 4:**
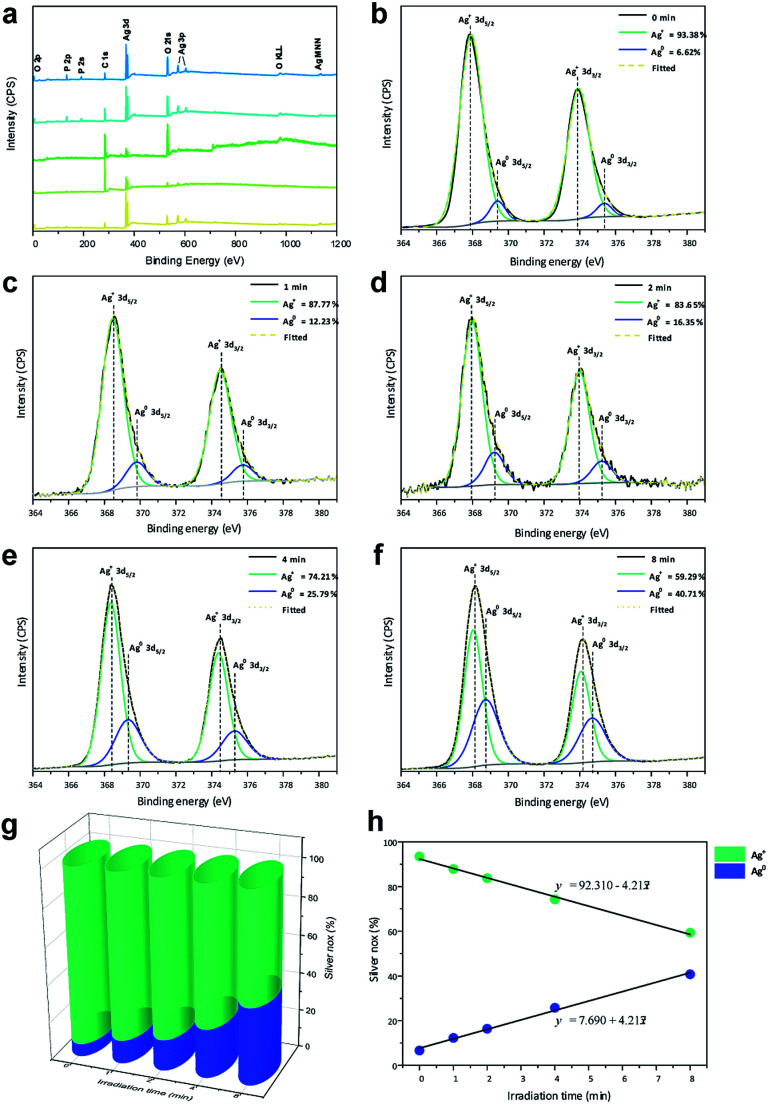
(a) XPS spectra and (b–f) high-resolution Ag 3d spectra of the samples; (g) contents of Ag^+^ and Ag^0^ for all samples and (h) linear formations of the different species.

XPS analysis was utilized to investigate the changes in chemical environment and binding energies by the electron irradiation of the pellets and define the valence states on the surfaces of the pellets. Ag, P, and O binding energy peaks were observed for all samples (Fig. 4a), in addition to C-related peaks attributed to the XPS instrument. Notably, no peaks related to other elements were identified, which confirms that the materials were composed only of Ag, P, and O.

Fig. 4b–f shows high-resolution XP spectra of Ag in the 3d region containing Ag 3d_3/2_ (373.93 eV) and Ag 3d_5/2_ (367.91 eV) doublets. For each peak, the deconvolution yielded two components, indicating two different oxidation states. For Ag^0^, the peaks were observed at 375.37 and 369.41 eV, corresponding to the 3d_3/2_ and 3d_5/2_ spin–orbit couplings, respectively. For Ag^+^, the peaks were observed at 373.93 and 367.91 eV corresponding to the 3d_3/2_ and 3d_5/2_ spin–orbit couplings, respectively.

The high-resolution spectra of Ag were used to quantify the amounts of Ag^0^ and Ag^+^ on the surfaces of the pellets, as shown in Fig. 4g. A small amount of Ag^0^ was observed in Ag_3_PO_4_ obtained without irradiation, as any type of electromagnetic radiation can reduce the Ag in the Ag_3_PO_4_ semiconductor. Upon irradiation of the material with electrons, a linear increase in Ag^0^ content and linear decrease in Ag^+^ content (Fig. 4h) were observed, which were used to obtain the formation equations for both of them as functions of the time. Based on these results, the relationship between the time and ratio between Ag^0^ and Ag^+^ in the Ag_3_PO_4_ pellet can be estimated, enabling a fine control of the surface composition.

Theoretical analysis to provide further insights into the atomistic transformations induced by the electron injection, AIMD simulations were carried out on the most favorable Ag_3_PO_4_ surfaces. Table 1 shows the characteristics of the surfaces. The (110) surface is the most stable and exhibits [AgO_2_] and [AgO_3_] under-coordinated clusters, whereas the (100) and (111) surfaces exhibit the under-coordinated [AgO_4_] cluster and over-coordinated [AgO_5_] cluster. Notably, the density of exposed Ag cations is also higher at the (110) surface, 11.7 cations per nm^2^ compared to 8.8 and 8.7 cations per nm^2^ at the (100) and (111) surfaces, respectively.

**Table 1:** Characteristics of the Ag_3_PO_4_ surfaces^a^

Surface	*E*_sur_ (J m^−2^)	Area (Å^2^)	Ag atoms per nm^2^	CN
(100)	1.85	145.2	8.8	[AgO_4_], [AgO_5_]
(110)	1.32	205.3	11.7	[AgO_2_], [AgO_3_]
(111)	1.51	251.4	8.7	[AgO_4_], [AgO_5_]

a CN = coordination number.

**Fig. 5:**
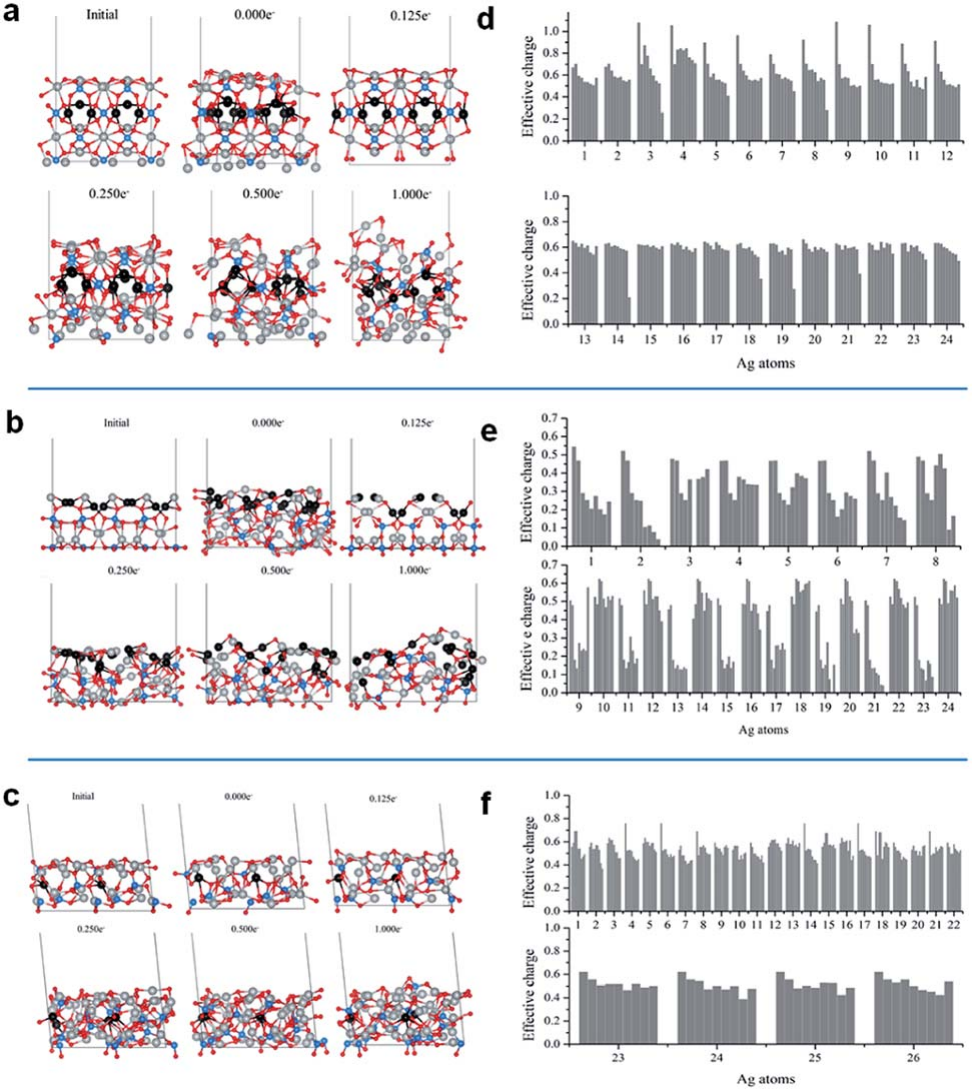
Lateral views of the initial and final configurations of the (a) 100, (b) 110, and (c) 111 surface at electron doses of 0.000, 0.125, 0.250, 0.500, and 1.000e^−^/Ag_3_PO_4_ unit; effective charges of the Ag cations in the outmost external layers of the (d) 100, (e) 110, and (f) 111 surface at the final configurations at electron doses of 0.000, 0.125, 0.250, 0.375, 0.500, 0.750, 0.875, and 1.000e^−^/Ag_3_PO_4_ unit.

Fig. 5a–c show the representative structures of each system at different electron doses. For all surfaces, a significant ordering is observed at an electron dose of 0.125e^−^. The disorder–order transition induced by the electron injection is consistent with the experimental XRD and Raman spectroscopy observations, *i.e.*, once the cubic metallic Ag is formed from Ag_3_PO_4_, a loss of semiconductor symmetry in the Raman spectrum is observed.

The additional electron dose yielded different structural modifications. The (110) surface exhibited more significant transformations. In Fig. 5b, the gray and black balls represent the Ag cations and Ag cations initially in the second surface layer, respectively. At the (110) surface, with the increase in electron dose, the Ag cations from the second layer moved upward. This process was accompanied by significant atom rearrangements leading to a surface termination with a high number of under-coordinated Ag cations. This surface did not exhibit considerable modifications upon the EBI, as shown in Fig. 5a and c. The local coordination of the Ag cations at the exposed surface also exhibited structural distortions, but most of the Ag cations remained in their original layers.

To understand the electron density distribution for each injection process, we analyzed the electronic structures of the representative configurations. Fig. 5d–f present the effective charges of the most superficial Ag cations at different electron doses. Reduction occurs with the increase in number of added electrons, which is more abrupt for the (110) surface, in which many Ag cations are strongly reduced even at the first dose and most of them have charges smaller than 0.2e^−^ at the last addition. For the other two surfaces, the superficial Ag cations are less severely reduced.

The analysis of the Ag–Ag and O–O distances in the representative configurations during the simulations showed structural rearrangements. These distances were reduced to the typical values of metallic Ag clusters and molecular O_2_ (Table S1†). In particular, a significant number of small Ag–Ag distances were observed for the (110) surface, while small O–O distances were observed for the (100) and (111) surfaces. To complement these theoretical results, we employed the QTAIM method to evaluate the presence of bond critical points of these systems. They were compared with Ag bulk and O_2_ molecule using the same type of calculation. Fig. S1 (ESI)† shows some of the representative cases. In particular, at the different electron doses, Ag clusters were formed on top of the (110) surface, in which the Ag–Ag distances were approximately 2.8 Å and the electron density and Laplacian values were in good agreement with those computed for the Ag bulk system. On the other hand, on the (100) and (111) surfaces, the formation of metallic clusters was scarce and only individual Ag dimers were observed in some configurations. However, in these structures, O_2_ molecules were observed at the outmost positions, as shown in Fig. S2 (ESI).†

These first-principles simulations demonstrate that the electron injection causes structural rearrangements of the most stable Ag_3_PO_4_ surfaces. In particular, the (110) surface, which initially had a higher density of exposed Ag cations, exhibited a large structural transformation involving diffusion events of Ag cations from the second layer to the surface, increasing the number of under-coordinated Ag cations at the exposed surface. This structural rearrangement led to an enhanced reduction of Ag cations to metallic Ag. Furthermore, QTAIM analysis demonstrated the formation of Ag clusters in the simulations, where the Ag–Ag distances were comparable to those in the metallic Ag bulk. In contrast, for the (100) and (111) surfaces, the formation of Ag clusters was scarcer and individual Ag dimers were observed in some configurations. However, the formation of superficial O_2_ molecules was observed in these systems.

These theoretical results suggest that the observed metallic Ag nanostructures initially more rapidly formed at the (110) surface, generating active nucleation sites at which the subsequent growth proceeded. In contrast, the (100) and (111) surfaces, having large amounts of oxygen anions and Ag coordination numbers higher than that of the (110) surface, exhibited a different process initiated by oxygen loss through O_2_ formation.

## 3. Conclusion and outlook

Major technological advancements are driven by the designs and development of equipment and materials with large potential to accelerate the development of various disciplines in chemistry, engineering, materials science, and nanotechnology. We presented a new EBI equipment with several inherent advantages over the conventional methods (*e.g.*, TEM) such as the compact size, low cost of manufacturing, and simple and safe operation with high reproducibility, which enables us to study the effects of the irradiation dose on different types of materials. The synthesis of the thick Ag film by the EBI equipment on the Ag_3_PO_4_ semiconductor was carried out for the first time without reducing agents or solvent. The interaction between these materials and electron beam enabled the migration of Ag cations from the crystal lattice to the surfaces, where the reduction of the positively charged Ag cations in the corresponding Ag metallic species occurred with the concomitant formation of the thick Ag film. These results provided valuable insights into the structures of the formed thick Ag film at the nanoscale and demonstrated the suitability of the employed processing method for the formation of thick films of metallic Ag on Ag_3_PO_4_. To support these findings and elucidate the formation of the thick Ag film at the atomic scale, detailed FE-SEM, XRD, Raman spectroscopy, and XPS analyses were carried out in combination with the AIMD simulations. The temporal evolutions of the calculated structures demonstrated the role of the local coordination of the Ag cations at the exposed surface and structural and electronic disorder-to-order transition processes.

Our simulations revealed that the surface density of the exposed Ag cations determined the metallic Ag growth. The process was initiated at the (110) surface where the Ag cations with a low coordination number were rapidly reduced forming metallic Ag clusters, which might eventually coalesce. At the (100) and (111) surfaces, where the densities of exposed Ag cations were lower, the reductions were slower and were accompanied by the formation of molecular O_2_ with the concomitant increase in number of exposed Ag cations.

This study suggests that the structure of thick Ag metal film–Ag_3_PO_4_ semiconductor is more complex than perceived. The effect of the time-dependent electron-beam-induced Ag layer formation at the semiconductor surface should be considered in following studies in the field of photo-electrochemistry with metal oxide semiconductors. We believe that our strategy paves the way for a low-cost and highly scalable manufacturing process for next-generation innovative materials and can guide the experimental syntheses of metal/semiconductor materials by the developed EBI equipment.

It is important to recognize that there are several open questions. It is crucial to identify the proper combinations of materials and develop measurement techniques to elucidate the unique structures created and the electronic states. The substrate is critical for the tuning of the properties of the as-synthetized nanocomposites. Further studies are required to understand the substrate effects on the device performances. In addition, the control of the size and shape of the thick film of metal nanoparticles on the surface is mandatory for scaling and thus should be considered in following studies.

## 4. Experimental

### Synthesis of Ag_3_PO_4_ pellets

Ag_3_PO_4_ semiconductors were synthesized by coprecipitation at 60 °C. First, 6 mmol of silver nitrate (AgNO_3_, Cennabras, 98%) and 1 mmol of diammonium hydrogen phosphate ((NH_4_)_2_HPO_4_, Alfa-Aesar, 98%) were dissolved in 50.0 mL of distilled water and 50.0 mL of ethanol in two beakers. After the dissolution, the solution of AgNO_3_ was added to the (NH_4_)_2_HPO_4_ solution and mixed for 20 min under magnetic stirring. The yellow precipitate was washed until the neutral pH was achieved and oven-dried at 60 °C. The Ag_3_PO_4_ semiconductor powder was then cold-pressed at a pressure of ≈500 MPa into circular pellets with diameters and thicknesses of 5 and 1 mm, respectively. Three pellets were prepared for each EBI to evaluate the reproducibility of the developed system and its use in the characterization techniques.

### EBI equipment

The developed device has several inherent advantages over the conventional methods such as the compact size, low cost of manufacturing, wide and uniform electron beam, possibility of operation under low and high vacuum levels, and insertion of gases through a flow control system. In addition, the device enables a simple and safe operation with a high reproducibility. The electron beam system includes a high-voltage power source, electron accelerator, and a chamber with vacuum system. The energies of the electrons are increased in one or more stages as they pass through the different acceleration anodes. The system includes three circular anodes (low-, medium-, and high-voltage accelerations) of nonmagnetic stainless-steel material designed with a central aperture for the uniform acceleration of electrons to the sample.

The voltage of the acceleration anodes can be adjusted with the low voltage (LV) anode between 0 V to 600 V, medium voltage (MV) anode from 1 kV to 3 kV and the high voltage (HV) anode from 10 kV to 25 kV. The cathode consists of a nonmagnetic stainless-steel material in a tapered shape to focus the electrons produced by the W filament to the sample. The system is controlled by a software. The high voltages applied to the three accelerators, the electron beam current, filament current, heating and cooling units for the specimen holder, gas injection system (comprising a mass flow controller), and vacuum are controlled to ensure the reproducibility of the system.

Along this experiment, the Ag_3_PO_4_ pellets were subjected to irradiation by the developed portable EBI equipment and the acceleration anodes was set at 600 V (LV), 3 kV (MV), 18 kV (HV) and current of 15 mA for 1, 2, 4, and 8 min. EBI at different times were used to observe the interactions of the electrons in the growth of Ag^0^ on the surfaces of the Ag_3_PO_4_ pellets. All the experiment of the irradiation was carried out under low vacuum (10^−3^ mTorr).

### Characterization and computational analysis

#### Scanning electron microscopy (FE-SEM)

In order to investigate the morphological characteristics of the prepared irradiated pellets and as a function of the total electron dose, it was used a field emission scanning electron microscope (FE-SEM), FEI (Model Inspect F50) operating at 5 kV. In this work the samples were cut mechanically for the cross-section measurements using FE-SEM images and the support image processing and analysis software (Image J). Mechanical cutting to perform cross-section measurements of the metallic layer was used, since cutting the relative Ag thick layer by more advanced techniques, such as the Focused Ion Beam (FIB), would induce silver growth, amorphization and deposition of gallium ions during the milling process, modifying the structure of the material.^70–74^

#### X-ray diffraction (XRD)

The crystal structure and long-range ordering of all prepared pellets were characterized by X-ray diffraction (XRD) in a D/Max-2500PC diffractometer (Rigaku, Japan) using Cu Kα radiation (*λ* = 1.54056 Å), in range of 10–110° 2*θ* at a scan rate of 0.5° min^−1^.

#### Micro-Raman scattering spectroscopy

Micro-Raman scattering spectroscopy measurements were employed in order to investigate the structural ordering of short range of the pristine and irradiated graphite pellets using a Bruker (model Senterra) spectrometer with an excitation laser of 785 nm (wavelength) and operating at a power of 50 mW.

#### X-ray photoelectron spectroscopy (XPS)

X-ray photoelectron spectroscopy measurements were employed in order to determine the surface composition of all pellet's samples using XPS (Scienta Omicron ESCA^+^, Germany), was carried out with monochromatic Al K_α_ radiation (1486.7 eV). The binding energies of all elements were calibrated by referencing to the C 1s peak at 284.8 eV.

#### Computational methods and model systems

First-principles total energy calculations were carried out within the periodic density functional theory framework using the Vienna *ab initio* simulation package (VASP).^75^ We used the Perdew–Burke–Ernzerhof D3 dispersion-corrected exchange–correlation functional, based on the generalized gradient approximation. A plane-wave basis set was used to describe the valence electrons.^76^ The projector augmented wave method^77^ was used to describe the interactions between the core and valence electrons.^78^ An energy plane-wave cut-off of 550 eV was used to achieve convergence of at least 1 meV per atom. Brillouin zone integrations were carried out using converged Monkhorst–Pack grids and tetrahedron method with Bloch corrections. The atomic Bader charges were computed using the quantum theory of atoms in molecules (QTAIM).^79^

Slab models of the (100), (110), and (111) surfaces were constructed using the bulk structure with lattice parameters obtained by previously reported calculations (*a* = *b* = *c* = 6.024 Å).^80^ Each slab model consisted of 16Ag_3_PO_4_ formula units (128 atoms), which were optimized prior to the AIMD simulations using electronic and ionic convergence criteria of 10^−4^ and 10^−2^ eV Å^−1^, respectively. The electron addition into the system was carried out using the NELECT flag implemented in VASP. The reported electron doses correspond to numbers of electrons per formula unit of Ag_3_PO_4_.

The AIMD simulations were carried out according to the following protocol: first, each surface model was thermalized at 300 K for 1 ps to ensure that the system reached thermal equilibrium. Several simulations at the same temperature were then carried out for 1 ps. At each stage, the following electron doses were introduced in the initial configuration: 0.000, 0.125, 0.250, 0.375, 0.500, 0.750, 0.875, and 1.000e^−^. The structural evolution was monitored during the simulation to identify representative configurations and characteristic events.

## Conflicts of interest

There are no conflicts to declare.

## Supplementary Material

RA-010-D0RA03179H-s001
