# Supplementary material for: Electron beam irradiation for the formation of thick Ag film on Ag3PO4
Source: RSC Adv. 2020 Jun 8;10(37):21745–53. doi: 10.1039/d0ra03179h (PMC9054597; doi:10.1039/d0ra03179h)
Supplement: RA-010-D0RA03179H-s001 [file RA-010-D0RA03179H-s001.pdf]

Electronic Supplementary Material (ESI) for RSC Advances.
This journal is © The Royal Society of Chemistry 2020

Table S1. Representative Ag clusters from the (110) surface upon four electron irradiation doses (0.125, 0.250, 0.500 and 1.000 e^-). Ag-Ag distances (Å) and the related ρ (a.u.) and $\nabla^2\rho$ (a.u.) are compared to Ag-Ag bonding in Ag bulk.

**System**	$\mathbf{d_{Ag\text{-}Ag}}$	ρ	$\nabla^2\rho$
Cluster 1 – 0.125 e^-	2.845	0.230	1.572
	2.744	0.274	2.346
	2.845	0.230	1.572
	2.744	0.274	2.346
Cluster 2 – 0.250 e^-	2.710	0.310	2.242
	2.869	0.233	1.510
	2.859	0.234	1.574
	2.882	0.209	1.579
Cluster 3 – 0.500 e^-	2.702	0.308	2.470
	2.842	0.224	1.820
Cluster 4 – 1.000 e^-	2.837	0.242	1.719
	2.822	0.264	1.454
	2.739	0.283	2.351
Metallic Ag	2.880	0.218	1.592

Table S2. Representative O_2 molecules formed in (100) and (111) surfaces upon two electron irradiation doses (0.125 and 0.250 e^-). O-O distances (Å) and the related ρ (a.u.) and $\nabla^2\rho$ (a.u.) are compared to O-O bonding in O_2 gas phase.

**System**	$\mathbf{d_{O\text{-}O}}$	ρ	$\nabla^2\rho$
(100) - 0.125 e^-	1.380	2.308	-7.575
(111) - 0.250 e^-	1.541	2.659	-12.124
Molecular O_2	1.234	3.435	-23.764

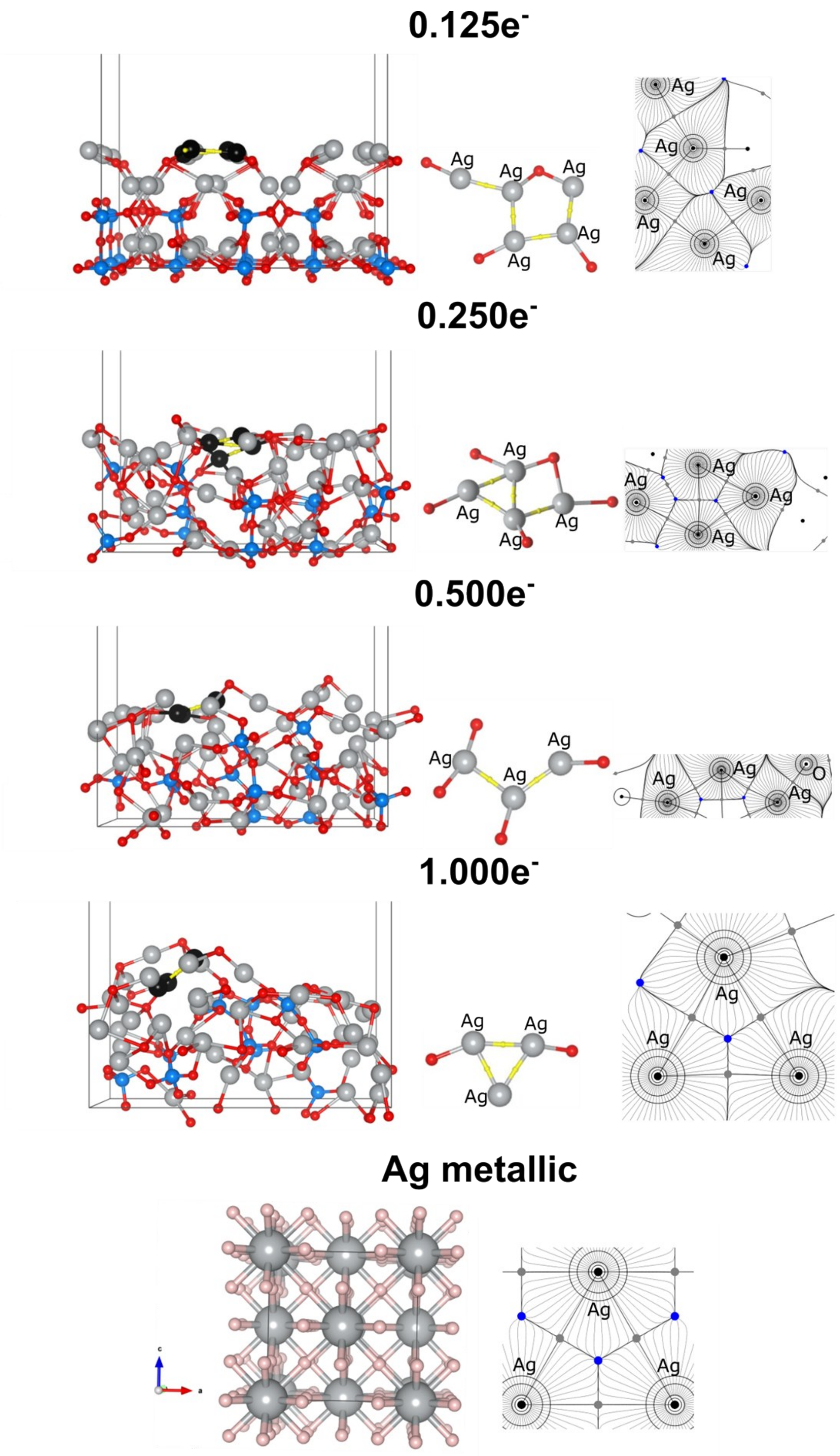

Figure S1. Representative Ag clusters from the (110) surface upon four electron irradiation doses (0.125, 0.250, 0.500 and 1.000 e^-). Left panels: system lateral views, where Ag, P and O atoms are coloured in grey, blue and red, respectively. The Ag atoms in the selected cluster are in black. Middle panels: top views of the selected Ag clusters. Right panels: illustrative representations of the bonding critical points (BCPs) of the selected Ag clusters.

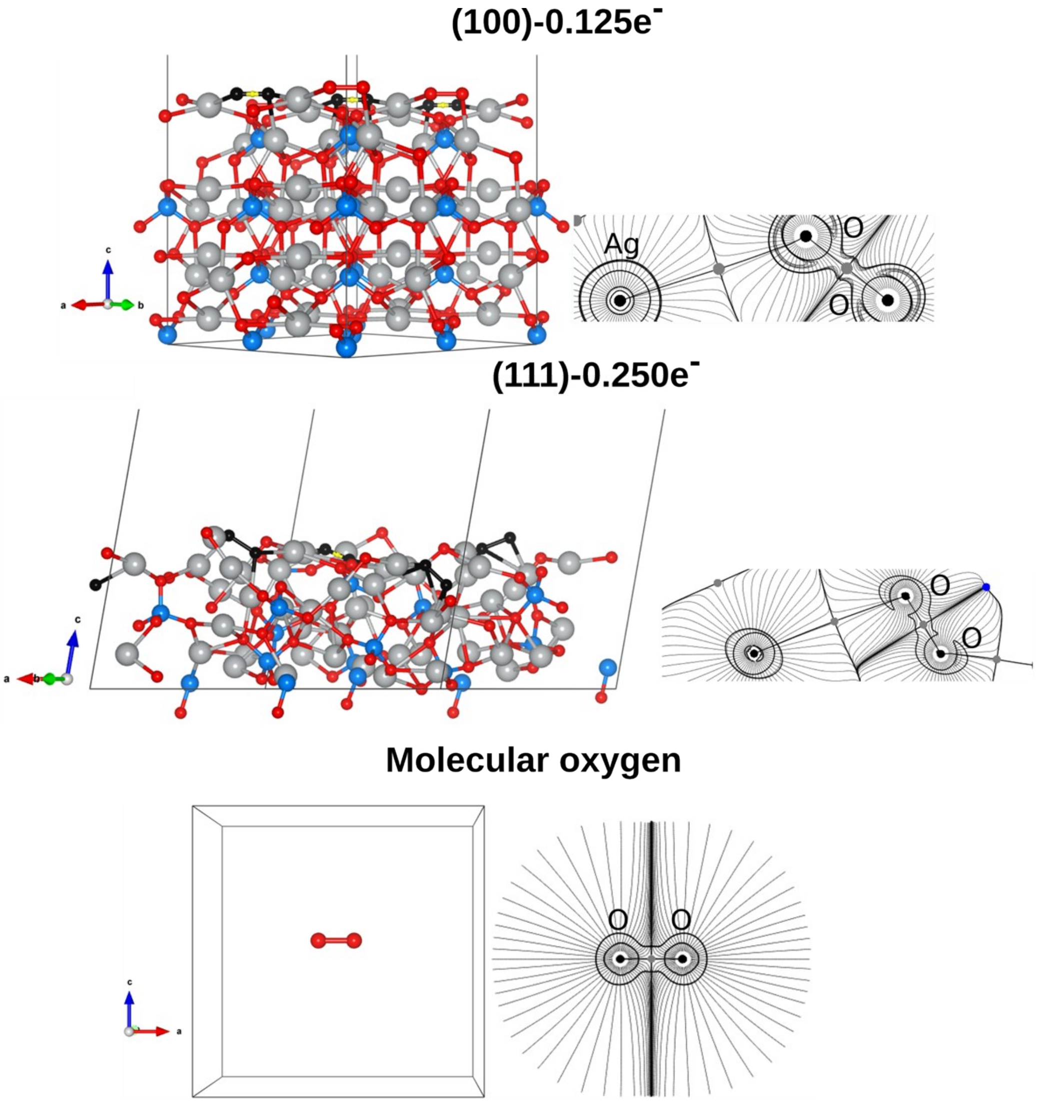

Figure S2. Representative O_2 molecules formed in (100) and (111) surfaces upon two electron irradiation doses (0.125 and 0.250 e^-). Left panels: system lateral views, where Ag, P and O atoms are coloured in grey, blue and red, respectively. The O atoms in the formed O_2 molecules are in black. Right panels: illustrative representations of the bonding critical points (BCPs) of the selected formed O_2 molecules.
